# Inverse association between soy food consumption, especially fermented soy products intake and soy isoflavone, and arterial stiffness in Japanese men

**DOI:** 10.1038/s41598-018-28038-0

**Published:** 2018-06-25

**Authors:** Hirokazu Uemura, Sakurako Katsuura-Kamano, Mariko Nakamoto, Miwa Yamaguchi, Miho Fujioka, Yuki Iwasaki, Kokichi Arisawa

**Affiliations:** 1 Department of Preventive Medicine, Institute of Biomedical Sciences, Tokushima University Graduate School, 3-18-15, Kuramoto-cho, Tokushima, 770-8503 Japan; 2 Department of Public Health and Applied Nutrition, Institute of Biomedical Sciences, Tokushima University Graduate School, 3-18-15, Kuramoto-cho, Tokushima, 770-8503 Japan

## Abstract

Studies on the associations between soy food consumption and arterial stiffness are rare. The aim of the present study was to evaluate their associations in Japanese men. A total of 652 eligible men, aged 35–69 years, who underwent the measurement of brachial-ankle pulse wave velocity (baPWV) as an index of arterial stiffness were evaluated in this cross-sectional study. Information on their lifestyle characteristics, including dietary behavior, was obtained from a structured self-administered questionnaire. The frequency of total soy products as well as fermented and non-fermented soy products intakes was calculated, and the amounts of soy protein and soy isoflavone intakes were also estimated; these were then divided into tertiles and their associations with baPWV values were evaluated using general linear models. Higher frequency of fermented soy products intake was associated with decreased baPWV after adjusting for the multivariable covariates (*P* value for trend was 0.002, in Model 3). This association did not alter after further adjustment with a biomarker of systemic inflammation (serum high-sensitivity C-reactive protein (hs-CRP)) (*P* value for trend was 0.001, in Model 4). Total soy isoflavone consumption was also inversely associated with baPWV even after adjusting for multivariable covariates including serum hs-CRP (*P* value for trend was 0.043, in Model 4); however total soy protein consumption was not. These results demonstrated that greater consumption of soy food, especially fermented soy products and soy isoflavone was associated with reduced arterial stiffness, independent of systemic inflammation, in Japanese men.

## Introduction

Since cardiovascular diseases are major causes of death in developed countries, early detection of cardiovascular damage is desired earnestly to prevent mortality and morbidity from cardiovascular diseases. Atherosclerotic changes in arteries mainly contribute to the pathogenesis of cardiovascular diseases, and increased arterial stiffness is demonstrated to be closely associated with atherosclerosis. Arterial stiffness can be evaluated by measuring arterial pulse wave velocity (PWV). Brachial-ankle PWV (baPWV) measurement is convenient, relatively quick, and reproducible. Moreover, baPWV value correlates well with the carotid-femoral PWV, which is an established index for assessing aortic stiffness^1^. Therefore, baPWV measurement has become popular in screening for arterial stiffness in Asian countries.

Isoflavone is a chemical compound that is structurally and biologically similar to estrogen^2^, and is known to have cardiovascular benefits. Dietary isoflavone is available through soybean and soy products. Soybean belongs to the legume family and is recognized as one of the plant foods. Soybeans have been a major component of the traditional Asian (including Japanese) diets, and are usually consumed as processed foods (tofu, natto, miso, shoyu, etc.). Fermented soy products such as miso and natto are often consumed in Japan as Japanese special food^3^. Soybean products are seen as healthy diets because of their beneficial nature of containing low fat, rich protein, rich vitamins, minerals, and fiber. Due to these beneficial nature and isoflavone content, soybean and soy products are considered to have potential cardiovascular benefits. However, the findings of studies evaluating the effects of soy food or soy isoflavone intake on cardiovascular benefits have been inconsistent^4–7^.

The antioxidative activity of fermented soybean products has been reported to be significantly higher than that in non-fermented steamed soybean^8,9^. Such antioxidative activity receives special attention because of its potential beneficial effect on cardiovascular health. However, reports on the relationships between the consumption of soy food products, especially the consumption of fermented soy products and soy isoflavone, and arterial stiffness which can detect early cardiovascular damage are few. The present study evaluated the possible relationships between frequency of intake of the total soy products, especially fermented soy products, and arterial stiffness using baPWV as the parameter in Japanese men. We also evaluated the relationships between the consumption of soy protein and soy isoflavone and arterial stiffness.

## Materials and Methods

### Study subjects

This cross-sectional study included men aged 35–69 years who participated in the baseline survey of a prospective cohort study from November 2009 to June 2012 in Tokushima Prefecture, Japan and who received a baPWV measurement at the baseline survey. The subjects were workers, and most of them were office workers and not shift workers. This study was performed as part of the Japan Multi-Institutional Collaborative Cohort (J-MICC) Study, a prospective cohort study. Details of this cohort study have been reported elsewhere^10^. Briefly, the J-MICC Study aims to examine the relationships of lifestyle and genetic factors as well as their interactions with lifestyle-related diseases.

All the participants in the J-MICC Study provided written informed consent prior to participation. The committees of Nagoya University School of Medicine, Aichi Cancer Center, and Tokushima University Graduate School approved the study protocol. This study was conducted according to the principles of the Declaration of Helsinki, and all methods were performed in accordance with the relevant guidelines and regulations.

### Questionnaire and evaluation for soy product consumption

Information on individual medical histories and lifestyle characteristics, including dietary behavior over the past year, was obtained through a structured self-administered questionnaire. All the responses were reviewed by trained staff at the time of the survey. Leisure-time exercise was estimated on the basis of the International Physical Activity Questionnaire^11^. Exercise was divided into three levels as follows: light (e.g., walking and hiking), moderate (e.g., light jogging and swimming), and vigorous (e.g., marathon running and competitive sports). The degrees of leisure-time exercise for the three levels were expressed as metabolic equivalent (MET)-hours/week (MET level × hours of activity × events per week) and summed. In this estimation, light, moderate, and vigorous exercises were assigned with 3.4, 7.0, and 10.0 METs, respectively.

### Evaluation for soy product consumption

Diet assessment was performed using a validated short food frequency questionnaire (FFQ) in the baseline survey of the J-MICC Study^12–15^. This FFQ included questions about the intake of 47 varieties of foods and beverages over the past year. This included questions about the intake of four groups of soy products: (Group1) miso soup; (Group2) tofu (soybean curd) for hiyayakko, yu-dofu; (Group3) natto and soybeans (boiled beans, etc.); and (Group4) fried tofu paste, fried bean curd, and thick deep-fried tofu. Eight categories of the frequency of each soy product intake were obtained as follows: three or more times/day (21/week), twice/day (14/week), once/day (7/week), 5–6 times/week (5.6/week), 3–4 times/week (3.5/week), 1–2 times/week (1.4/week), 1–3 times/month (0.7/week), and never or seldom (0/week). The total frequency of soy products intake was calculated as the sum of the frequencies of the four soy products intakes. According to the National Health and Nutrition Survey 2012^16^, Japanese population rarely consume soybeans compared to natto. Natto occupies the majority of intake in Group3 (natto and soybeans). Miso is Japanese fermented soybean paste, while natto is made from soybeans fermented with Bacillus subtilis. Hence, the frequency of fermented soy products intakes was calculated as the sum of the frequencies of Group1 (miso soup) and Group3 (natto and soybeans) intakes. The frequency of non-fermented soy products intakes was calculated as the sum of the frequencies of Group2 (tofu) and Group4 (fried tofu paste, fried bean curd, and thick deep-fried tofu) intakes.

Next, we estimated the amount of total soy protein and total soy isoflavone intakes, as previously reported^17^. Since there were no questions on the size of each soy product consumed in the FFQ, 3-day diet records were surveyed four times in approximately 3 months’ intervals (in four seasons in Japan) within one year, in a group of 28 participants. This was used to grasp the serving size of each soy product per meal in our population to estimate the weekly amounts of total soy protein and total soy isoflavone intakes. The portion sizes of (1) miso soup, (2) tofu, (3) natto, soybean, and (4) fried tofu paste, fried bean curd, and thick deep-fried tofu were 9.9 (the amount used as miso), 53.8, 31.7, and 19.5 (g/meal), respectively. The amount of total soy protein intake was estimated by summing the soy protein contained in each specific soy food on the basis of the Standard Tables of Food Composition in Japan 2010^18^. The amount of the total soy isoflavone intake was estimated by summing the soy isoflavone contained in each soy food based on the estimates of the phytoestrogens in foods in Japan^19^.

Daily intake of total energy was estimated using a program developed by the Department of Public Health, Nagoya City University School of Medicine^12,13^.

### Measurements

baPWV was measured using a waveform analyzer (model BP-203RPE III; Colin, Co. Ltd., Komaki, Japan), as described previously^20^. In brief, a subject was examined while resting in the supine position in an air-conditioned room. Extremity blood pressure was measured using an oscillometric method, and the ankle brachial index (ABI) was automatically calculated. baPWV was calculated through a time-phase analysis between the right brachial artery pressure and volume waveforms at both ankles. To reduce inter-observer variations, all baPWV measurements were performed by a single researcher throughout the duration of the study. Individual baPWV and ABI data were expressed as the means of the bilateral baPWV and ABI, respectively.

Body mass index (BMI) was calculated as weight (kg) divided by height (m) squared. Venous blood was aspirated from each participant, and serum was separated within 3 hours and stored at −80 °C. Biochemical factors, including lipids and high-sensitivity C-reactive protein (hs-CRP) as a biomarker of systemic inflammation, in the stored sera, were measured at an external laboratory (BML Inc., Tokyo, Japan).

### Statistical analyses

**Figure 1:**
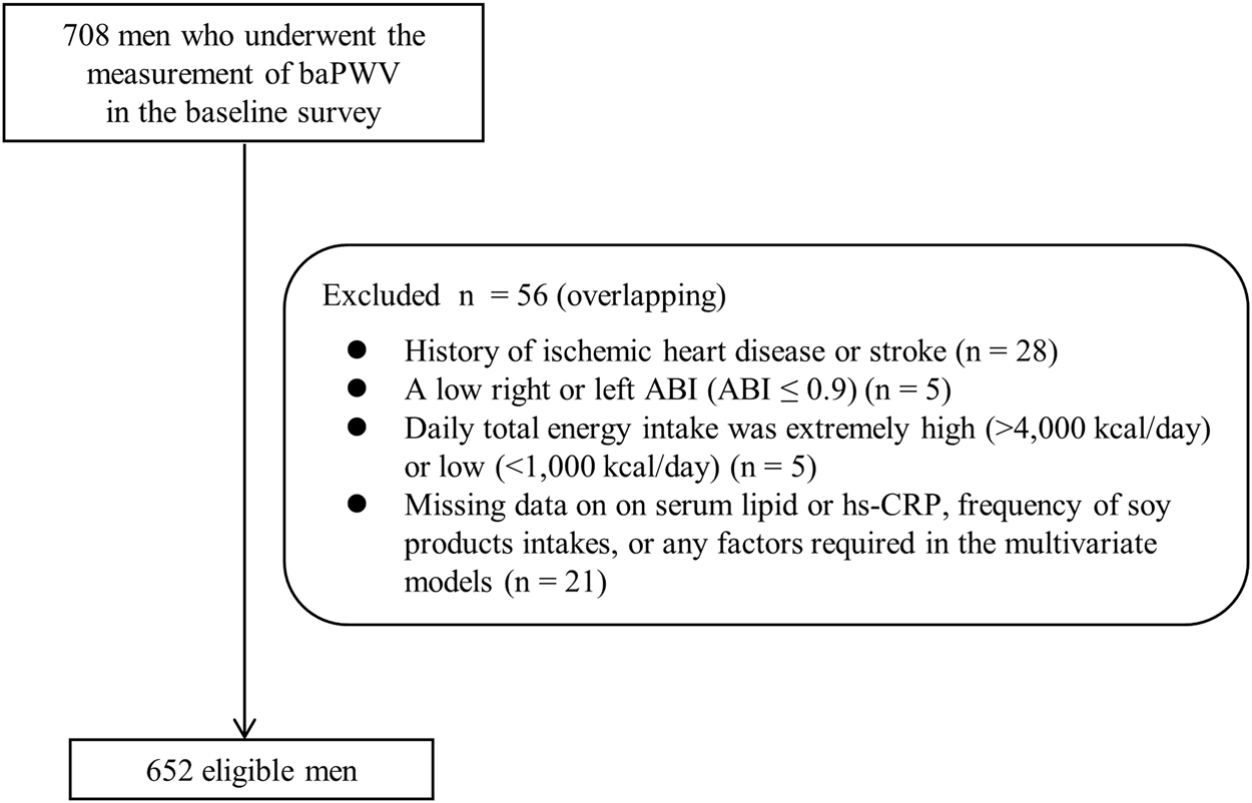
A flow chart of the analyzed subjects. From 708 men who underwent the measurement of baPWV in the baseline survey, we finally analyzed 652 eligible men. baPWV, brachial-ankle pulse wave velocity; ABI, ankle-brachial pressure index.

Of the 708 men initially included in this cross-sectional study, we excluded in total, 56 men (with overlapping characteristics) as follows (shown in Fig. 1): (1) 28 men with a history of ischemic heart disease or stroke; (2) 5 men with a low right or left ABI (ABI ≤ 0.9), which suggested peripheral arterial occlusive disease; (3) 5 men whose estimated daily total energy intake was extremely high (>4,000 kcal/day) or low (<1,000 kcal/day); and (4) 21 men who had no data on serum lipid or hs-CRP, frequency of soy products intakes, or any factors required in the multivariate models. Data on a total of 652 men were finally analyzed.

The total frequency of soy products intakes was divided into tertiles; the lowest category was used as the reference. Continuous variables were expressed as mean ± standard deviation (SD) or median values (25th percentile, 75th percentile). Categorical variables were expressed as numbers (%). The analysis of variance, Kruskal-Wallis test, or chi-square test was used to compare the characteristics between the tertile categories of the total frequency of soy products intakes, where appropriate.

General linear models were used to evaluate the relationships of frequency of total, fermented, and non-fermented soy products intakes (tertiles) as well as the amount of total soy protein and total soy isoflavone intakes (tertiles) with baPWV after adjusting for the covariates in four model groups. In Model 1, age (continuous) and systolic blood pressure (5 categories: <120, 120 to <140, 140 to <160, or ≥160 mmHg without medical treatment, and use of antihypertensive agent), which are recognized as being very closely associated with arterial stiffness were adjusted for. In Model 2, adjustment was made for age, systolic blood pressure, BMI (kg/m^2^, quartiles), smoking habit (current or others), alcohol drinking (current or others), exercise (MET-hours/week, quartiles), hypercholesterolemia (≥220 mg/dL or receiving medical treatment, no/yes) low high-density lipoprotein (HDL) cholesterol (<40 mg/dL, no/yes), elevated triglyceride levels (≥150 mg/dL, no/yes), diabetes (receiving medical treatment, no/yes), and daily energy intake (kcal/day, quartiles). In Model 3, the covariates adjusted for in Model 2 as well as coffee intake (≤1 cup/day, >1 to 3 cups/day, and >3 cups/day), calcium intake (mg/day, continuous, log-transformed) and total fiber intake (g/day, continuous, log-transformed) which might be associated with baPWV were adjusted for. Moreover, in Model 4, the covariates adjusted for in Model 3 as well as serum hs-CRP levels (mg/L; quartiles) were adjusted for. baPWV values and total fiber intake showed right skewed distributions and were included in the analyses after logarithmic transformation. Tests for trends were performed by assigning the ordinal variables of 1, 2, and 3 for each tertile category.

All calculations and statistical tests were performed using SAS version 9.4 (SAS Institute Inc., Cary, NC, USA). Statistical tests were based on 2-sided probabilities, and the level of significance was set at *P* < 0.05.

## Results

The mean age (SD) and median BMI (25^th^, 75^th^) of the study subjects were 48.8 (8.6) years and 24.2 (22.3, 26.1) kg/m^2^, respectively. The median baPWV (25^th^, 75^th^) was 1,404 (1274, 1563) cm/s.

### Characteristics of the subjects according to the frequency of total soy products and fermented soy products intakes

Tables 1 and 2 show the respective characteristics of the subjects according to the frequency of total and fermented soy products intakes. Subjects with greater frequency of total, as well as fermented soy products intakes showed higher degree of leisure-time exercise, higher energy intake, and greater consumption of calcium, total fiber, total soy protein, and total soy isoflavone, and lower prevalence of current smoking. Subjects in the highest tertiles of total as well as fermented soy products intakes showed the highest mean ages. baPWV values were marginal-significantly different between the tertiles of the frequency of fermented soy products intake (*P* value was 0.080), and these values gradually decreased as the frequency of fermented soy products intake increased.

**Table 1:** Characteristics of the subjects according to the frequency of total soy products intake.

	Frequency of total soy products intake (times/week)			*P*
	T1 (≤4.9)	T2 (>4.9 to 8.4)	T3 (>8.4)	
Number (%)	232 (35.6)	203 (31.1)	217 (33.3)	
Age (years)^a^	47.0 ± 8.4	48.8 ± 8.7	50.7 ± 8.4	<0.001
Body mass index (kg/m^2^)^b^	24.1 (22.4, 26.0)	24.6 (22.2, 26.7)	23.9 (22.1, 25.4)	0.068
Current smoking, n (%)				
No	135 (58.2)	136 (67.0)	157 (72.4)	0.006
Yes	97 (41.8)	67 (33.0)	60 (27.6)	
Current alcohol drinking, n (%)				
No	53 (22.8)	61 (30.0)	57 (26.3)	0.234
Yes	179 (77.2)	142 (70.0)	160 (73.7)	
Exercise (MET-hours/week)^b^	2.80 (0.43, 14.53)	4.70 (0.43, 15.05)	5.25 (1.28, 15.30)	0.025
Systolic BP (mmHg)^a^	135.0 ± 16.5	134.5 ± 17.3	134.7 ± 15.8	0.944
Diastolic BP (mmHg)^a^	84.1 ± 11.6	84.3 ± 11.6	84.7 ± 10.7	0.812
Prevalence, n (%)				
Hypertension	104 (44.8)	93 (45.8)	105 (48.4)	0.740
Hypercholesterolemia	104 (44.8)	76 (37.4)	87 (40.1)	0.280
Low HDL cholesterol	14 (6.0)	11 (5.4)	7 (3.2)	0.357
Elevated triglycerides	66 (28.4)	54 (26.6)	68 (31.3)	0.557
Diabetes	7 (3.0)	10 (4.9)	13 (6.0)	0.312
Energy intake (kcal/day)^b^	1743 (1530, 1920)	1788 (1641, 1950)	1876 (1702, 2067)	<0.001
Coffee intake (cups/day)^b^	1.65 (1.5, 3.5)	1.65 (1.5, 3.0)	1.65 (0.8, 3.5)	0.089
Calcium intake (mg/day)^b^	355 (312, 467)	414 (364, 498)	458 (387, 554)	<0.001
Total fiber intake (g/day)^b^	7.3 (6.3, 8.6)	8.3 (7.3, 9.5)	9.9 (8.4, 11.9)	<0.001
Intake frequency of total soy products (times/week)^b^	3.5 (2.8, 4.2)	7.0 (6.3, 7.7)	11.2 (9.8, 14.7)	<0.001
Intake frequency of fermented soy products (times/week)^b^	2.1 (1.4, 2.8)	4.9 (4.2, 6.3)	8.4 (7.0, 10.5)	<0.001
Intake frequency of non-fermented soy products (times/week)^b^	1.4 (0.7, 2.1)	1.4 (1.4, 2.1)	2.8 (2.1, 4.2)	<0.001
Total soy protein consumption (g/week)^b^	8.5 (5.9, 10.9)	14.2 (11.8, 17.4)	27.8 (19.9, 38.3)	<0.001
Total soy isoflavone consumption (mg/week)^b^	31.5 (18.7, 41.3)	55.6 (45.3, 65.3)	102.3 (78.3, 151.6)	<0.001
Serum hs-CRP (mg/L)^b^	0.36 (0.22, 0.86)	0.37 (0.19, 0.81)	0.34 (0.18, 0.73)	0.380
ABI^a^	1.12 ± 0.07	1.12 ± 0.06	1.13 ± 0.06	0.118
BaPWV (cm/s)^b^	1429 (1272, 1574)	1399 (1281, 1581)	1404 (1251, 1540)	0.710

^a^Mean ± SD. ^b^Median (25%, 75%).

BP, blood pressure; HDL, high density lipoprotein; hs-CRP, high-sensitivity C-reactive protein.

ABI, ankle-brachial pressure index; baPWV, brachial-ankle pulse wave velocity.

**Table 2:** Characteristics of the subjects according to the frequency of fermented soy products intake.

	Frequency of fermented soy products intake. (times/week)			*P*
	T1 (≤2.8)	T2 (>2.8 to 6.3)	T3 (>6.3)	
Number (%)	211 (32.4)	215 (33.0)	226 (34.7)	
Age (years)^a^	48.0 ± 8.5	47.8 ± 8.9	50.5 ± 8.3	0.001
Body mass index (kg/m^2^)^b^	24.1 (22.4, 25.9)	24.4 (22.5, 26.7)	24.0 (22.0, 25.7)	0.193
Current smoking, n (%)				
No	131 (62.1)	134 (62.3)	163 (72.1)	0.040
Yes	80 (37.9)	81 (37.7)	63 (27.9)	
Current alcohol drinking, n (%)				
No	53 (25.1)	49 (22.8)	69 (30.5)	0.165
Yes	158 (74.9)	166 (77.2)	157 (69.5)	
Exercise (MET-hours/week)^b^	2.98 (0.43, 15.30)	3.90 (1.28, 11.70)	5.95 (1.28, 15.30)	0.026
Systolic BP (mmHg)^a^	135.3 ± 16.2	134.5 ± 17.5	134.5 ± 15.7	0.868
Diastolic BP (mmHg)^a^	84.4 ± 11.5	84.2 ± 11.6	84.5 ± 10.8	0.973
Prevalence, n (%)				
Hypertension	98 (46.4)	96 (44.7)	108 (47.8)	0.803
Hypercholesterolemia	99 (46.9)	73 (34.0)	95 (42.0)	0.023
Low HDL cholesterol	12 (5.7)	11 (5.1)	9 (4.0)	0.701
Elevated triglycerides	61 (28.9)	58 (27.0)	69 (30.5)	0.712
Diabetes	7 (3.3)	8 (3.7)	15 (6.6)	0.192
Energy intake (kcal/day)^b^	1758 (1536, 1923)	1770 (1588, 1966)	1852 (1698, 2052)	<0.001
Coffee intake (cups/day)^b^	1.65 (1.5, 3.5)	1.65 (1.5, 3.5)	1.65 (0.8, 3.5)	0.192
Calcium intake (mg/day)^b^	378 (318, 486)	405 (355, 479)	450 (377, 548)	<0.001
Intake frequency of total soy products (times/week)^b^	3.5 (2.1, 4.2)	6.3 (5.6, 7.7)	11.2 (9.1, 14.7)	<0.001
Intake frequency of fermented soy products (times/week)^b^	1.4 (1.4, 2.1)	4.9 (4.2, 5.6)	7.7 (7.0, 10.5)	<0.001
Intake frequency of non-fermented soy products (times/week)^b^	1.4 (0.7, 2.1)	1.4 (0.7, 2.1)	2.1 (1.4, 4.2)	<0.001
Total fiber intake (g/day)^b^	7.3 (6.3, 8.6)	8.2 (7.3, 9.5)	9.5 (8.3, 11.7)	<0.001
Total soy protein consumption (g/week)^b^	9.3 (5.9, 12.8)	14.2 (10.4, 17.4)	24.8 (17.8, 37.9)	<0.001
Total soy isoflavone consumption (mg/week)^b^	34.9 (18.7, 49.5)	55.0 (39.9, 67.9)	97.0 (69.0, 147.8)	<0.001
Serum hs-CRP (mg/L)^b^	0.33 (0.20, 0.85)	0.40 (0.19, 0.86)	0.35 (0.20, 0.72)	0.718
ABI^a^	1.12 ± 0.07	1.12 ± 0.06	1.13 ± 0.06	0.169
BaPWV (cm/s)^a^	1437 (1302, 1581)	1400 (1267, 1575)	1386 (1251, 1528)	0.080

^a^Mean ± SD. ^b^Median (25%, 75%).

BP, blood pressure; HDL, high density lipoprotein; hs-CRP, high-sensitivity C-reactive protein.

ABI, ankle-brachial pressure index; baPWV, brachial-ankle pulse wave velocity.

### Relationships between the frequency of total, fermented, and non-fermented soy products intakes and arterial stiffness

Table 3 shows the adjusted associations of the frequency of total, fermented, and non-fermented soy products intakes with baPWV (analyzed by general linear models). Higher frequency of total soy products intakes was associated with decreased baPWV after adjusting for the multivariable covariates including coffee, calcium, and total fiber intake (*P* value for trend was 0.005, in Model 3). This association did not alter after further adjustment with serum hs-CRP (*P* value for trend remained 0.005, in Model 4).

**Table 3:** Associations of the frequency of total, fermented, and non-fermented soy products intakes with baPWV.

	Intake frequency (times/week)						*P* for trend
	T1		T2		T3		
	Adjusted mean	(95%CI)	Adjusted mean	(95%CI)	Adjusted mean	(95%CI)	
Total soy products	T1 (≤4.9)		T2 (>4.9 to 8.4)		T3 (>8.4)		
Model 1	1494	(1469, 1518)	1469	(1444, 1493)	1436	(1412, 1460)	<0.001
Model 2	1523	(1478, 1570)	1505	(1459, 1552)	1468	(1424, 1514)	<0.001
Model 3	1522	(1476, 1570)	1506	(1460, 1553)	1469	(1424, 1516)	0.005
Model 4	1520	(1473, 1568)	1503	(1457, 1550)	1467	(1422, 1514)	0.005
Fermented soy products	T1 (≤2.8)		T2 (>2.8 to 6.3)		T3 (>6.3)		
Model 1	1495	(1470, 1521)	1474	(1450, 1499)	1432	(1409, 1456)	<0.001
Model 2	1527	(1481, 1575)	1513	(1467, 1560)	1467	(1424, 1512)	<0.001
Model 3	1526	(1479, 1574)	1514	(1467, 1561)	1469	(1425, 1514)	0.002
Model 4	1525	(1478, 1573)	1510	(1464, 1558)	1466	(1422, 1512)	0.001
Non-fermented soy products	T1 (≤0.7)		T2 (>0.7 to 2.1)		T3 (>2.1)		
Model 1	1478	(1450, 1508)	1459	(1438, 1479)	1471	(1444, 1499)	0.755
Model 2	1502	(1454, 1552)	1494	(1451, 1539)	1506	(1459, 1554)	0.820
Model 3	1496	(1448, 1546)	1494	(1450, 1538)	1512	(1465, 1561)	0.402
Model 4	1493	(1445, 1543)	1491	(1448, 1536)	1510	(1462, 1559)	0.396

baPWV, brachial-ankle pulse wave velocity; T1, first tertile; T2, second tertile; T3, third tertile; CI, confidence interval.

Model 1: adjusted for age and systolic blood pressure.

Model 2: adjusted for age, systolic blood pressure, body mass index, smoking habit, alcohol drinking, exercise, hypercholesterolemia, low high-density lipoprotein cholesterol, elevated triglyceride levels, diabetes, and daily energy intake.

Model 3: adjusted for the covariates in model 2 plus coffee intake, calcium intake, and total fiber intake.

Model 4: adjusted for the covariates in model 3 plus serum hs-CRP levels.

baPWV values were log-transformed before analyses, and geometric adjusted means and their 95% CIs are presented.

Further analyses categorizing soy products into fermented or not fermented, revealed that the frequency of fermented soy products intakes was inversely associated with baPWV after adjusting for multivariable covariates including serum hs-CRP (*P* value for trend was 0.001, in Model 4). However, the frequency of non-fermented soy products intakes was not associated with baPWV.

### Relationships of total soy protein and total soy isoflavone consumption with arterial stiffness

As shown in Table 4, total soy isoflavone consumption was inversely associated with baPWV even after adjusting for multivariable covariates including serum hs-CRP (*P* value for trend was 0.043, in Model 4). In contrast, the inverse association between total soy protein consumption and baPWV attenuated, becoming non-significant when adjusting for coffee, calcium, and total fiber intake (Model 3), or additionally, serum hs-CRP (Model 4).

**Table 4:** Associations of the estimated intake amount of total soy protein and total soy isoflavone with baPWV.

	Estimated intake amount						*P* for trend
	T1		T2		T3		
	Adjusted mean	(95%CI)	Adjusted mean	(95%CI)	Adjusted mean	(95%CI)	
Total soy protein (g/week)	T1 (≤11.4)		T2 (>11.4 to 18.5)		T3 (>18.5)		
Model 1	1492	(1467, 1517)	1459	(1435, 1484)	1447	(1422, 1471)	0.005
Model 2	1521	(1475, 1569)	1495	(1450, 1541)	1480	(1435, 1526)	0.016
Model 3	1518	(1470, 1566)	1495	(1450, 1542)	1484	(1438, 1532)	0.092
Model 4	1514	(1467, 1563)	1493	(1448, 1540)	1482	(1436, 1530)	0.112
Total soy isoflavone (mg/week)	T1 (≤41.5)		T2 (>41.5 to 70.2)		T3 (>70.2)		
Model 1	1495	(1470, 1520)	1458	(1434, 1483)	1445	(1420, 1469)	0.002
Model 2	1525	(1479, 1573)	1493	(1448, 1539)	1478	(1433, 1525)	0.006
Model 3	1523	(1475, 1571)	1493	(1448, 1539)	1481	(1435, 1529)	0.037
Model 4	1519	(1472, 1568)	1491	(1446, 1537)	1479	(1433, 1527)	0.043

baPWV, brachial-ankle pulse wave velocity; T1, first tertile; T2, second tertile; T3, third tertile; CI, confidence interval.

Model 1: adjusted for age and systolic blood pressure.

Model 2: adjusted for age, systolic blood pressure, body mass index, smoking habit, alcohol drinking, exercise, hypercholesterolemia, low high-density lipoprotein cholesterol, elevated triglyceride levels, diabetes, and daily energy intake.

Model 3: adjusted for the covariates in model 2 plus coffee intake, calcium intake, and total fiber intake.

Model 4: adjusted for the covariates in model 3 plus serum hs-CRP levels.

baPWV values were log-transformed before analyses, and geometric adjusted means and their 95% CIs are presented.

## Discussion

The current study revealed that greater consumption of total soy products, especially fermented soy products and soy isoflavone, was dose-dependently associated with decreased arterial stiffness, independent of traditional atherosclerotic risk factors or systemic inflammation, in Japanese men.

Since cardiovascular diseases are leading causes of death in developed countries, measures against cardiovascular damage is important to prevent mortality and morbidity from cardiovascular diseases. Soybean and soy products contain rich protein, vitamins, minerals, fiber, and isoflavone; therefore, they are paid special attention due to the potential cardiovascular benefits. Findings of a most recent meta-analysis concluded that soy consumption was negatively associated with the risk of cardiovascular diseases, stroke, and coronary heart disease risk^4^. However, in the subgroup meta-analyses, a statistically significant protective effect of soy consumption on cardiovascular disease risk was primarily observed in Asian populations^4^. In contrast, Low *et al*.^5^ in a meta-analysis combining cohort studies, showed no association between soy intake and the risk of stroke or coronary heart disease, although a significantly inverse association between soy intake and the risk of stroke and coronary heart disease was observed in case-control studies. Thus, currently, the conclusions for the favorable effect of soy intake on the risk of cardiovascular diseases have remained inconsistent.

Soybeans are usually consumed as processed foods (tofu, natto, miso, shoyu, etc.) in Japan, and fermented soy products such as miso and natto are Japanese special food^7^. Therefore, we additionally evaluated the associations between soy food consumption and arterial stiffness by categorizing soy food into fermented or not fermented. The frequency of fermented soy products intakes was inversely associated with arterial stiffness after adjusting for multivariable covariates, while frequency of non-fermented soy products intakes was not. In a population-based cohort study in Japan, natto intake was associated with the decreased risk of cardiovascular disease mortality^21^. Natto possesses strong fibrinolytic activity and anti-coagulation profiles^22,23^. Natto is also demonstrated to have favorable effect on blood pressure^24^. Antioxidative activity has potential beneficial effect on cardiovascular health, and the antioxidative activity of fermented soybean products has been reported to be significantly higher than that in non-fermented steamed soybean^8,9^. The findings of these reports lend support to the results of our study. The present study also revealed that intake of total soy isoflavone, which has antioxidative activity, was inversely associated with arterial stiffness even after adjusting for multivariable covariates. When estimated total isoflavone consumption was used as a continuous variable (mg/week) (not by tertiles), it was also inversely correlated with baPWV: age-adjusted rank order correlation coefficient between total soy isoflavone consumption and baPWV (continuous, cm/sec) was −0.147 (*P* < 0.001). These results are concordant with the results in a previous systemic review which reported that soy isoflavone supplementation provides an effective means of reducing arterial stiffness^25^. However, according to the previous reports in Europe, dietary isoflavone intake was not associated with cardiovascular disease risk in Dutch women^6^ or with cardiovascular disease mortality in Spanish adults^7^. Low *et al*.^5^ also reported in their meta-analysis that no association between soy isoflavone intake and the risk of stroke and coronary heart disease was identified. Currently, as similar to the effect of soy food intake, the effect of soy isoflavone intake on the cardiovascular risk have remained inconsistent. Possible explanations for these inconsistencies include the differences in study designs, different amounts of soy intake in target populations, and different types of consumed soy products. The amount of soy consumption, especially intake amounts of fermented soy products, is higher in Asian populations than in Western populations^26^. In addition, the prevalence of equol producer is higher in Asian populations (about 50–60%)^27^ than in Western populations (about 20–30%)^28^. Equol is a metabolite of the dietary isoflavone produced by the action of intestinal bacteria in response to soy isoflavone intake^27^. Equol has selective affinity for the estrogen receptor β, that is also expressed in the vasculature. Equol possesses a longer half-life and higher bioavailability than genistein and daidzein, being the major isoflavones in soybean, and equol has been suggested to have the highest antioxidant properties of the isoflavones^27,29^. Recently, equol producer status has been reported to be inversely associated with arterial stiffness in Japanese women around menopause and early postmenopause^30^ and coronary calcification in Japanese men^31^. Equol supplementation has also been reported to decrease arterial stiffness in men^32^ and women^33^. Then, equol may have greater anti-atherosclerotic properties than other isoflavones. Because fermented soy products can skip the initial steps of soy metabolism in the intestine, they may contribute to larger production of equol than non-fermented soy food. Further studies determining equol-producing abilities of the individuals will be needed to determine the relationships between soy isoflavone consumption and cardiovascular health.

Coffee is often consumed worldwide including Japan. Coffee contains rich antioxidants such as chlorogenic acid and other bioactive compounds^34^. Epidemiologic studies have demonstrated inverse associations between coffee intake and diabetes^35^ and metabolic syndrome^36,37^, which are high risk conditions for atherosclerosis. A previous study conducted by us demonstrated that coffee consumption was inversely associated with arterial stiffness in Japanese men^38^. Dietary calcium is also suggested to be inversely associated with cardiovascular risk and mortality^39,40^; however, excessive calcium intake via supplementation may have adverse influence on vascular events^41^. Calcium is considered to be involved in the regulation of vascular smooth muscle cell contractility^42^ and reducing platelet aggregation^43^. Sufficient dietary fiber intake is also demonstrated to be associated with a reduced risk of cardiovascular disease through its impact on the glycemic response^44,45^. Soybean products contain rich calcium and fiber. Therefore, we additionally adjusted for coffee intake, calcium intake, and total fiber intake, which may be associated with arterial stiffness in the analyses. The inverse associations between the frequency of fermented soy products intakes as well as soy isoflavone consumption and arterial stiffness were slightly attenuated but remained significant, although the association between soy protein consumption and arterial stiffness became non-significant. This finding suggests that fermented soy products as well as soy isoflavone intake may be associated with reduced arterial stiffness. This association may be slightly dependent on intakes of coffee as well as calcium and fiber which are rich in soy products, however, other independent mechanisms may exist.

Chronic systemic inflammation has been recognized as a key player in the pathogenesis of various diseases including cardiovascular disease^46,47^. Hs-CRP is a sensitive biomarker of systemic inflammation^48^, and hs-CRP measurement in blood has recently become popular in clinical and health examination settings for assessing low-grade systemic inflammation. After further adjusting for serum hs-CRP levels (Model 4), the inverse associations between the frequency of fermented soy products intakes as well as soy isoflavone consumption and arterial stiffness did not alter. Therefore, the observed associations with arterial stiffness might be independent of chronic systemic inflammation.

The present study has several limitations. First, because of the cross-sectional study design, causal relationships between soy products and soy isoflavone consumption and reduced arterial stiffness could not be established. Second, information about the frequency of soy products intakes was self-reported; therefore, non-differential misclassification might have been inevitable. Moreover, we could not obtain information on the size of the consumed foods. Hence, we calculated the intake of soy products per meal from a four, 3-day diet records of 28 participants, in order to estimate soy protein and isoflavone consumption. Correlation coefficients between the estimated consumptions of soy protein and isoflavone from the FFQs and the diet records were 0.47 and 0.59 (Spearman’s rank correlation), respectively^17^. Additionally, the amounts of soy protein and soy isoflavone intakes as well as the total energy intake may be underestimated due to our short FFQ; however, their ranking might be satisfactory. Third, we did not determine the equol-producing abilities of the subjects. Fourth, although we adjusted for a number of potential confounding factors in the analyses, residual confounding could not be eliminated. Fourth, our study included a relatively small number of subjects. Finally, because all of our subjects were Japanese men, the results may not be generalizable to women or to other ethnic populations.

In conclusion, our study demonstrated that greater consumption of soy products, especially fermented soy products, as well as soy isoflavone was associated with reduced arterial stiffness, independent of the classical atherosclerotic risk factors and of chronic systemic inflammation, in Japanese men. Prospective or interventional studies that additionally include women are required to confirm our findings.
